# Recent Developments in Lipid Nanoparticle-Mediated Delivery of Biotherapeutics and Gene Therapy Across the Blood–Brain Barrier

**DOI:** 10.1007/s40259-026-00782-0

**Published:** 2026-04-30

**Authors:** Umar Iqbal, Roy W. Hwang, Will J. Costain

**Affiliations:** 1 Human Health Therapeutics Research Centre, National Research Council of Canada, 1200 Montreal Road, Ottawa, ON K1A 0R6 Canada; 2 Department of Cellular Molecular Medicine, Faculty of Medicine, University of Ottawa, Ottawa, ON Canada

## Abstract

Peripherally administered therapeutics for neurological indications are challenged with anatomical and physiological barriers that limit their ability to access their site of action in the central nervous system (CNS). This is particularly true for complex therapeutics such as antibodies, immunotherapeutics, and gene therapies. The blood–brain barrier is the specialized structure that functionally regulates the ability of blood constituents to access the CNS. Blood–brain barrier delivery technologies for protein therapeutics have been established in pre-clinical models and are beginning to be verified in clinical studies. Technologies reliant on the transcellular pathway across the blood–brain barrier utilize the receptor-mediated transcytosis mechanism. Research into the use of lipid nanoparticles (LNPs) to deliver complex therapeutics has tremendously expanded in recent years. Lipid nanoparticles represent a compelling alternative to viral vectors for the delivery of various gene therapy modalities, including messenger RNA, small interfering RNA, and antisense oligonucleotides. Functionalization of LNPs with blood–brain barrier-penetrant moieties is being explored as a means to enable CNS delivery of LNP-based therapeutics. The recent innovations and validation of LNP-based delivery systems have hastened the fulfillment of the promise of facile CNS-targeted gene therapies. This review focuses on functional aspects of the blood–brain barrier and how they relate to recent advances in LNP technologies for CNS delivery, as well as their potential impact on gene therapy.

## Key Points

**Table:** 

The development and refinement of antibodies that exploit receptor-mediated transcytosis to deliver peripherally administered therapeutics to the central nervous system have enabled new treatment modalities for central nervous system diseases.
The recent clinical validation of the safety and utility of nanoparticle delivery systems has ignited research into the functionalization of lipid nanoparticles for targeted delivery. Similarly, recent developments in clustered regularly interspaced short palindromic repeats (CRISPR) technology have addressed many challenges and presented new opportunities for gene therapy.
The functionalization of lipid nanoparticles with receptor-mediated transcytosis ligands represents a timely solution to the challenge of delivering CRISPR-based gene therapies to the central nervous system.

## Lipid Nanoparticles: A Uniquely Capable Central Nervous System Therapeutic Modality

Solving the challenge of the blood–brain barrier (BBB) for central nervous system (CNS)-targeted therapeutics continues to be an area of intensive research focus. While directly bypassing the BBB using invasive methods (intracranial, intrathecal, or intracerebroventricular [ICV] administration) or temporarily disrupting the BBB (focused ultrasound or osmotic disruption) are viable strategies for a limited number of CNS delivery applications, they are associated with considerable safety and implementation issues [1, 2]. Strategies that focus on crossing the BBB by exploiting endogenous transport mechanisms, such as receptor-mediated transcytosis (RMT), aim to avoid the drawbacks of invasive methods [3–5]. Advances in nanotechnology have led to the development of nanoparticles (NPs) formulated for BBB permeability [4]. Among the various NP formats developed, lipid nanoparticles (LNPs) have undergone extensive clinical validation for safety and efficacy. Indeed, LNPs occupy a unique niche in a vast therapeutic landscape that encompasses small-molecule drugs with simple chemical structures, multi-functional protein biologics, nucleic acids, NPs, viruses, and genetically engineered cells. Each of these therapeutic modalities has its utility and limitations. Herein, we review recent advances in LNP formulation, functionalization, and CNS targeting from the perspective of BBB structure, function, and disease. Moreover, we highlight the versatility of LNP delivery vehicles as a uniquely positioned alternative to viruses in advancing CNS gene therapy.

## NPs and the BBB

The development of nanocarriers for drug delivery has become a highly active area of research. Concurrently, gene therapy applications, particularly those utilizing adeno-associated virus (AAV), hold great promise and have advanced to clinical trials [6]. As viruses (20–300 nm) and NPs (5–200 nm) exceed the size limit for diffusion across the BBB via the paracellular pathway, they must rely on the endocytic or transcytotic pathways [7]. While the majority of AAV serotypes are not capable of crossing the BBB, certain AAV serotypes exhibit brain tropism and are able to cross the BBB and subsequently transduce neurons [6, 8]. Numerous capsid engineering efforts have been undertaken to further exploit brain tropism and have resulted in further optimizations of brain delivery of AAV-mediated gene therapy [6, 9]. Notably, the recently identified AAVBR1 is an AAV2 variant reported to be highly specific for brain microvascular endothelial cells and may represent a means to locally produce therapeutic proteins or to genetically modulate the BBB to enhance its amenability to NP transport to the CNS [10, 11]. Mechanistically, AAV capsid proteins bind receptors present on brain endothelial cells (BECs) and subsequently undergo RMT in a manner that is distinct from their transduction mechanism [12–14].

Systemically administered NPs are largely excluded from the brain [15], necessitating the engineering of BBB-penetrant properties in nanomaterials to target CNS disorders [7, 16, 17]. While viruses as small as 25 nm can deliver meaningful therapeutic effects, the magnitude of therapeutic effect for NPs is size dependent. Larger NPs (> 100 nm) have greater drug loading capacity, whereas smaller NPs (< 100 nm) exhibit greater cellular uptake and prolonged circulation time [18]. Moreover, very small (< 10 nm) and very large (200–500 nm) NPs are rapidly cleared by the kidney, liver, and spleen [15, 19]. Given that high clearance rates are suboptimal for CNS delivery, optimizing NPs for CNS delivery requires careful attention to a variety of factors, including size, charge, and surface functionalization.

In comparison to viral vectors, LNPs offer several advantages for gene therapy applications. Viruses (adenovirus, AAV and lentivirus) are associated with immunogenicity, payload restrictions and potential genotoxicity due to insertional mutagenesis. Lipid nanoparticle-delivered gene therapy strategies can be designed to avoid these pitfalls while offering the capability to exceed what is possible with a viral vector. Moreover, the cost and complexity of manufacturing therapeutic viruses vastly exceed that of LNPs [20]. Thus, LNP-mediated CNS gene therapy represents a highly promising enabling technology for curative therapeutics targeting neurological disorders.

## LNPs for CNS Drug Delivery

Lipid nanoparticles represent an innovative advancement in CNS drug delivery, offering new potential to overcome the BBB and deliver diverse therapeutic cargoes directly to the brain. These sophisticated LNP platforms encompass various formulations, including solid lipid nanoparticles, nanostructured lipid carriers, traditional liposomes, and cutting-edge ionizable lipid systems [21]. Each platform offers distinct advantages: solid lipid nanoparticles provide excellent stability and controlled-release profiles, nanostructured lipid carriers offer enhanced drug loading capacity through their imperfect crystalline structure, liposomes enable biomimetic membrane interactions, and ionizable lipid systems facilitate endosomal escape through pH-dependent protonation. The versatility of LNP platforms is high, enabling encapsulation of an extensive array of therapeutic agents that range from small molecules and peptides to complex biologics, including proteins, antisense oligonucleotides (ASOs), small interfering RNA (siRNA), messenger RNA (mRNA), plasmid DNA, and clustered regularly interspaced short palindromic repeats (CRISPR)-Cas gene editing components [22, 23]. The encapsulation process for nucleic acids typically involves ionizable cationic lipids that form electrostatic complexes with the negatively charged payload at acidic pH during NP assembly. Nano-encapsulation protects therapeutic cargoes against in vivo enzymatic degradation and systemic clearance, thereby enabling targeted delivery. The focus of this review will be on systems that incorporate ionizable cationic lipids and their applicability to targeted CNS delivery.

## BBB Structure and Function

**Fig. 1:**
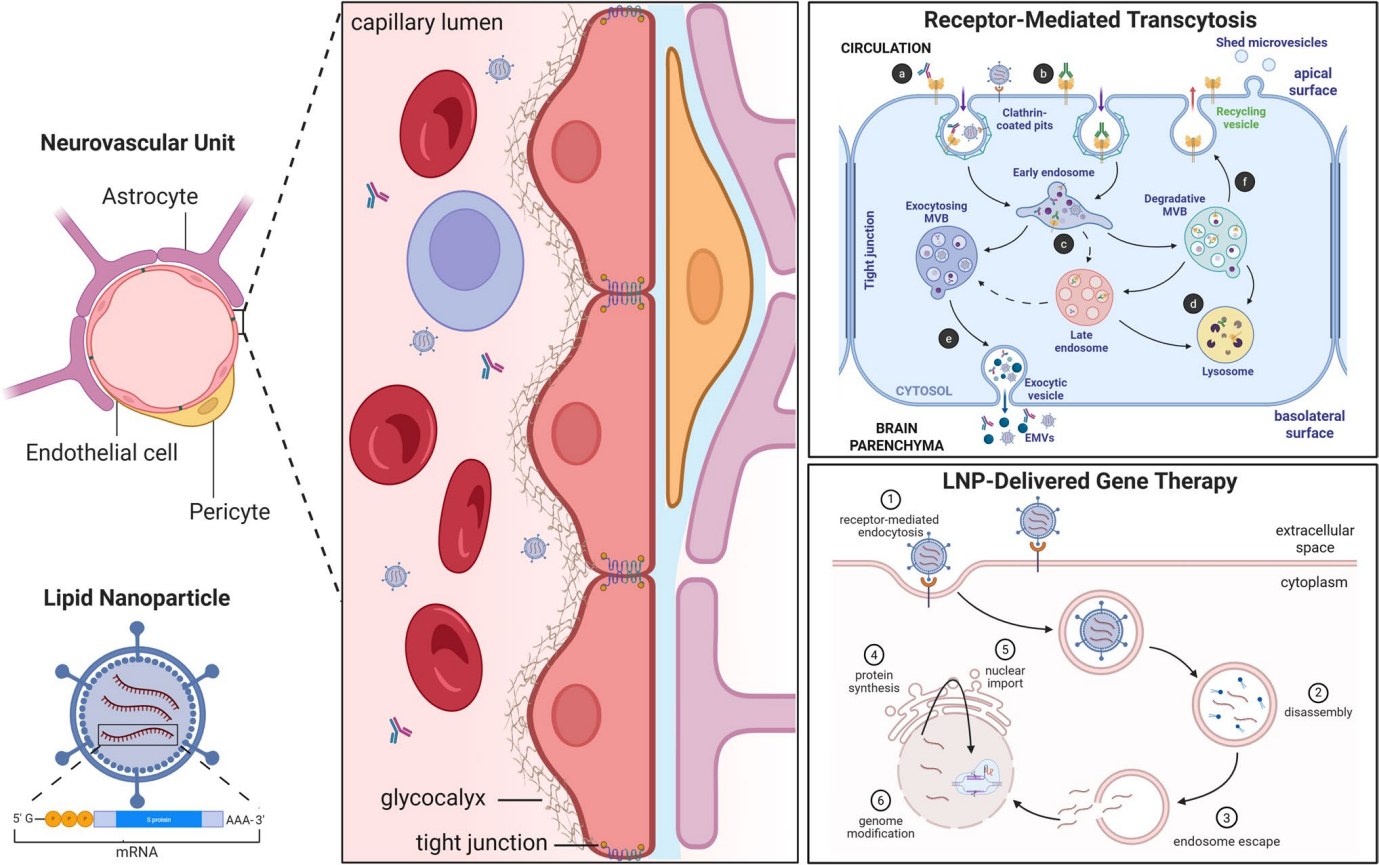
Schematic of the neurovascular unit structure, the mechanisms of blood–brain barrier transmigration, and lipid nanoparticle (LNP)-RNA delivery in cells. Adjacent brain endothelial cells form tight junctions owing to the presence of adhesion proteins within the plasma membrane. Exposure of therapeutics circulating in the blood to brain endothelial cells can be affected by the presence of the negatively charged glycocalyx. Antibodies and receptor-mediated transcytosis (RMT) ligand-functionalized LNPs can bind receptors, such as the transferrin receptor (TfR), present on the endothelial cell surface and undergo RMT (*top right panel*). **a** Monovalent antibodies or LNPs bind receptors on the luminal side (circulation) of a brain endothelial cell. **b** Bivalent antibody binding to the TfR on a brain endothelial cell. **c** Following clathrin-mediated endocytosis and sorting to the early endosome, antibody ligands or LNPs are further sorted to either exocytosing or degradative multivesicular bodies (MVBs). Dissociation of low-affinity/avidity antibodies can occur, in part due to the reduced endosomal pH (pH sensitivity). **d** High-affinity bivalent interactions that are not pH sensitive are thought to induce TfR dimerization and lead to trafficking toward degradative MVBs and ultimately lysosomal degradation of both the antibody and TfR. **e** Low-affinity, monovalent, or pH-sensitive binding results in trafficking to exocytosing MVBs and ultimately exocytic vesicles where LNPs and antibodies are released, either as free solutes or via exocytosing MVBs (EMVs), into the parenchyma (abluminal side). **f** Non-antibody-bound TfR are trafficked from degradative MVBs to recycling vesicles, where they are redisplayed on the brain endothelial cell surface. Alternatively, partial sorting to late endosomes and subsequently to EMVs may occur [27]. Targeted LNP delivery of gene therapy (*lower right panel*) occurs in cells expressing cell surface receptors that bind the internalization ligand displayed on the LNP. (1) The LNP ligand binds its cell surface receptor and initiates receptor-mediated endocytosis. (2) Endocytosed LNPs are trafficked to endosomes, where the LNP is disassembled into its constituent components. (3) The LNP cargo escapes the endosome and is released into the cytoplasm, where it is imported into the nucleus. (4) The messenger RNA (mRNA) undergoes export to the endoplasmic reticulum for clustered regularly interspaced short palindromic repeats (CRISPR) protein synthesis. (5) The CRISPR protein is then imported into the nucleus, where it can perform genome modifications (6) (created with BioRender.com)

Peripherally administered neurotherapeutics face a uniquely difficult challenge in accessing their targets in the CNS. This is largely owing to the BBB, which is a highly effective obstacle that uses multiple mechanisms to prevent select blood constituents (solutes and cells) from entering the brain [8]. The BBB is a component of the neurovascular unit (NVU; Fig. 1), which is an anatomical and functional structure that responds to the varying energy demands of the brain by regulating cerebral blood flow [24]. In addition to BECs, the NVU consists of astrocytes, mural cells (i.e., vascular smooth muscle cells and pericytes), microglia, neurons, and extracellular matrix components. The NVU is structured such that the interior of the microvasculature and capillaries is lined with endothelial cells that are attached to the extracellular matrix of the basement membrane (basal lamina) (Fig. 1). The luminal surface of the endothelial cells is covered with a mucosal layer (polysaccharide macromolecules known as the glycocalyx) that acts as a sieve-like barrier to large molecules and NPs [16]. The abluminal surface of the vessels is surrounded by pericytes and astrocytic end feet, and is in contact with neuronal projections. The multi-cellular NVU structure permits neurovascular coupling, which links neural activity and cerebral blood flow, enabling precise spatiotemporal regulation of blood flow that corresponds to localized energy demands [24]. Importantly, astrocytes and pericytes play a well-documented role during the development and maintenance of the BBB [25], and the barrier properties of the BBB rely on the cellular crosstalk between pericytes, astrocytes, and neurons within the NVU [26].

Blood constituents within the lumen of the capillaries are directly exposed to the endothelial cell layer of the NVU. As such, BECs represent the first and primary opportunity to restrict access to the brain. Brain endothelial cells are specialized tight junction (TJ)-forming endothelial cells that effectively restrict paracellular transport of hydrophilic proteins > 500 Da across the BBB [28], which is enhanced by the low rate of pinocytosis of BECs [29, 30]. The intercellular TJs are formed from rows of extensive overlapping occlusions between BECs, with structural integrity being provided by TJ protein complexes [29]. Tight junction complexes are formed by a collection of proteins, including occludins, claudins, junctional adhesion molecules, and ZO-1, with claudins considered to be the principal component in establishing TJs and determining the paracellular barrier between adjacent BECs [31]. Because of the negative charge of the glycocalyx, molecules that are positively charged readily interact with the glycocalyx, whereas negatively charged molecules are repelled [16]. The importance of the glycocalyx in mediating the uptake/transport of LNP nanocarriers was recently demonstrated by Szecskó et al. [32]. These authors showed that altering the surface charge of endothelial cells, by neuraminidase or cationic lipid treatment, significantly reduced uptake of cationic peptide-tagged NPs. Thus, electrostatic interactions must be considered when designing brain-targeted therapeutics.

In addition to the physical TJ barrier, BECs are capable of actively transporting specific solutes across the BBB. This is accomplished by the expression of numerous mono-directional or bi-directional transporter proteins that regulate the transport of specific solutes into or out of the brain [33]. In the case of nutrients, such as glucose, amino acids, nucleosides, and certain neurotransmitters, entry into the brain is accomplished through specific solute transporters such as glucose transporter 1, monocarboxylate transporter 1 (SLC16A1), and large neutral amino acid transporter (LAT1, SLC7A5) [28, 33, 34]. Conversely, efflux transporters, such as the ABC transporters, pump solutes from the brain into the blood, thereby clearing harmful metabolites, as well as playing a role in preventing blood solutes from entering the brain [33–35]. The efflux transporter system is responsible for many small-molecule therapeutics that exhibit lower CNS permeability than would be expected from their lipophilicity [35, 36]. An additional component of the BBB is a metabolic barrier wherein drug-metabolizing enzymes inactivate therapeutics and/or alter their ability to enter the CNS [29]. While the protection the brain receives from potentially harmful blood constituents is necessary for brain function, the BBB also prevents many therapeutics from engaging their CNS targets.

## Mechanisms of CNS Drug Delivery

Complex therapeutics (e.g., macromolecules, NPs, viruses) targeting CNS pathologies must either be directly administered to the brain or rely on a capacity to be delivered to the brain from the peripheral vasculature [8]. A variety of strategies, including clinically implemented medical procedures such as mannitol-induced osmotic BBB disruption [37], and microbubbles with focused ultrasound [2, 38], have been developed to overcome the BBB and enable delivery of therapeutics into the brain. While BBB disruption mechanisms can effectively enable brain permeation in a limited set of diseases, their physical nature is inherently damaging and is not tenable for diseases requiring repeated long-term dosing [16]. In contrast, transcytotic mechanisms are not associated with BBB disruption or invasive delivery of blood constituents. Transcytosis can be mechanistically subdivided into carrier-mediated transport, RMT, or adsorptive-mediated transcytosis (AMT). The stereospecific transport of smaller cargoes (e.g., glucose, hormones, and fatty acids) is achieved by carrier-mediated transport, while AMT enables the transport of positively charged molecules by virtue of electrostatic interactions with cell surface binding sites [16]. Receptor-mediated transcytosis has proven to be a robust mechanism for delivering a wide variety of therapeutic modalities across the BBB, with multiple receptor systems exploitable for this purpose [8].

Endocytosis is the underlying mechanism for AMT and RMT, and involves internalization of extracellular molecules into intracellular vesicular structures (endosomes). Endosomes are highly heterogeneous, and can be broadly classified as early/sorting endosomes, recycling endosomes, and late endosomes [27, 39, 40]. Classification has been based on size, morphology, cellular localization, and protein expression. Expression of protein markers is used to differentiate between early/sorting endosomes (Rab5a, Eea1), recycling endosomes (Rab4, Rab11), and late endosomes (Lamp 1/2, M6pr, Rab7a, Rab11a/b) [27, 39]. Nanoscale intraluminal vesicles within the early endosome are formed by the invagination of the limiting membrane of the early endosome, resulting in the creation of multivesicular bodies (MVBs) [41]. These intermediate endosomal structures transition to either degradative MVBs (late endosomes and lysosomes) [42] or exocytosing MVBs, which ultimately form microvesicles that are shed to the extracellular environment [43]. The ability of BECs to shed exocytosing MVBs to their abluminal surface represents the enabling mechanism of RMT (summarized in Fig. 1).

Endocytosis can be classified as clathrin-mediated endocytosis or clathrin-independent endocytosis, including caveolin-dependent endocytosis and pinocytosis. Clathrin-mediated endocytosis (also known as receptor-mediated endocytosis) is a selective process that requires binding to a cell-surface receptor as the first step in the internalization of a specific subset of extracellular molecules. Receptor-mediated endocytosis is a complex controlled process involving numerous factors in the plasma membrane and cytosol [44]. Clathrin-mediated endocytosis and RMT are similar processes, with RMT occurring in a restricted subset of polarized cell types, including neurons, epithelial cells, and endothelial cells [44, 45]. The establishment and maintenance of apicobasal polarity in BECs rely on the function of intercellular tight junctions [46], and BEC function is dynamically regulated by numerous factors, including sheer stress and growth factor signaling [47]. Receptor-mediated transcytosis in barrier-forming cells (epithelial and endothelial cells) is the underlying mechanism responsible for the transport of select macromolecules (such as iron, insulin, and insulin-like growth factors) across the BBB. The initiation of RMT and clathrin-mediated endocytosis is identical, namely the binding of a ligand to a receptor expressed on the luminal (apical) surface of the BEC. Clathrin-mediated endocytosis-mediated internalization of the ligand-receptor complex is followed by sorting into early endosomal vesicles. Multivesicular body formation ensues, with ligands sorted into degradative, recycling, or exocytosing MVBs. Ligands present in exocytosing MVBs are released to the abluminal (basolateral surface, transport to brain parenchyma) or luminal (apical surface, recycling to vessel lumen) sides of the endothelial cell. Notably, most AAV serotypes use glycan moieties (heparan sulfate for AAV2,3,6; N-linked and O-linked sialic acid for AAV1,4,5,6, and terminal galactose for brain trophic AAV9) for initial attachment and engage the AAV receptor KIAA0319L and various co-receptors post-attachment [48–50]. Similarly, brain trophic AAV variants have been found to utilize GPI-linked receptors (lymphocyte antigen 6A, lymphocyte antigen 6C1) or transmembrane proteins (carbonic anhydrase 4, LDL receptor-related protein 6, transferrin receptor (TfR, TFRC), alkaline phosphatase) independently of KIAA0319L to effect brain trophism and BBB transcytosis [14, 50, 51]. Mechanistically, KIAA0319L enables intracellular trafficking (retrograde transport from endosomes to the trans-Golgi network), is required for transduction, and is likely to utilize the above-described mechanism for transcytosis. Similarly, functionalizing NPs to bind known endothelial transcytosis receptors (TfR, insulin receptor, transmembrane protein 30A, insulin-like growth factor 1 receptor [IGF1R], low-density lipoprotein receptor, low-density lipoprotein receptor-related protein 1 [LRP1], CD98, basigin, integrin-beta 1, integral membrane protein 2A, neonatal Fc receptor, P-selectin [SELP]) represents a viable strategy for enabling CNS delivery of therapeutic NPs to the brain parenchyma following peripheral administration [5, 16].

Additionally, there are alternative, less well-understood transcytosis mechanisms, such as AMT [52] and caveolar transcytosis [53], both of which exploit electrostatic interactions between cationic or biomimetic NP surfaces and endothelial membranes to bypass barriers. Adsorptive-mediated transcytosis relies on non-specific electrostatic interactions between positively charged NPs and the negatively charged endothelial glycocalyx, triggering macropinocytosis and subsequent transcellular transport [54]. Caveolar transcytosis utilizes caveolin-1-mediated vesicular transport, which is upregulated in BECs during diseased conditions and avoids the lysosomal degradation pathway [53].

## BBB and Neuropathology

It has become increasingly evident that structural alterations to the NVU and BBB dysfunction are common observations in numerous neurological diseases and injuries associated with inflammation and neurodegeneration [16, 26]. Blood–brain barrier dysfunction has been observed in animal models and patients with Alzheimer’s disease (AD) [55], Parkinson’s disease (PD) [56, 57], Huntington’s disease (HD) [58–60], amyotrophic lateral sclerosis [61–63], and multiple sclerosis [64, 65], as well as other diseases. Indeed, it is estimated that >40% of brain disorders exhibit dysfunction and/or structural alterations to the BBB [26]. Clinically, BBB dysfunction can be observed using dynamic contrast enhanced-magnetic resonance imaging, by the presence of circulating biomarkers such as claudin-5, occluding, and ZO-1, or by examining the cerebrospinal fluid (CSF)/serum albumin ratio [26]. Post-mortem analysis of brain tissue showing the presence of blood components (fibrinogen, immunoglobulin G, albumin) or pathological immune cell trafficking into the brain parenchyma indicates compromised BBB in neuropathological conditions [26]. Currently, the primary mechanism thought to cause disease-related BBB dysfunction is an altered TJ structure and decreased expression of TJ proteins [25, 26]. Blood–brain barrier dysregulation and disruption under disease conditions represent an underappreciated aspect of neuropathology that may facilitate drug delivery [16], as well as an opportunity for developing BBB-targeted therapeutics that could restore the essential function of the BBB.

## BBB and Blood–Brain Tumor Barrier Modulation for CNS Gene Delivery

The BBB imposes a major obstacle to CNS drug delivery under physiological conditions, and this limitation persists in glioblastoma (GBM) through the blood–brain tumor barrier, which remains heterogeneously and often incompletely disrupted [66]. While BBB integrity is compromised within hypoxic and angiogenic tumor cores, the blood–brain tumor barrier stays largely intact in low-grade gliomas and at infiltrative margins of high-grade tumors, where glioma cells migrate into adjacent brain tissue without an overt barrier breakdown [66]. This regional barrier continuity contributes to therapeutic resistance and tumor recurrence. Blood–brain tumor barrier properties vary with tumor grade: low-grade gliomas largely preserve BBB-like microvascular architecture, whereas high-grade GBMs exhibit increased vascular surface area, vessel diameter, and permeability that exceed those of normal white matter [66]. Spatial heterogeneity in pericyte coverage, tight junction protein expression (e.g., claudin-5, occludin), and endothelial transcytosis further drives differential permeability and drug accumulation across tumor compartments [67].

To overcome these challenges, LNP-based strategies have been developed that harness ligand-mediated targeting of receptors such as TfR or LRP1, mannose-mediated immune cell uptake, and biomimetic NP coatings to improve penetration and cellular specificity [66, 68]. Complementary to physiological targeting, several physical modulation approaches, including focused ultrasound, optical (light-based) stimulation, and magnetic field-based strategies, enable transient and region-specific increases in BBB and blood–brain tumor barrier permeability [69–72]. Among these, focused ultrasound combined with circulating microbubbles has demonstrated the greatest clinical translatability, achieving a reversible and localized barrier opening that enhances CNS delivery of nucleic acid-loaded LNPs. In preclinical GBM models, this approach increased NP accumulation up to approximately ten-fold in healthy brain regions and approximated six- to seven-fold within tumors while preserving vascular and neurological integrity [69].

Light-based BBB modulation primarily exploits near-infrared photothermal or optogenetic mechanisms to alter endothelial junctions and barrier function with high spatial precision [70, 71]. Near-infrared-absorbing nanomaterials such as gold nanorods generate localized hyperthermia upon irradiation, sufficient to perturb TJ integrity and increase paracellular permeability while limiting widespread tissue damage [70]. Optogenetic platforms expressing light-responsive ion channels or modulating RhoA signaling in endothelial cells have demonstrated reversible control of barrier permeability in in vitro and ex vivo models [71]. Magnetic field-mediated strategies utilize superparamagnetic iron oxide NPs or magnetoelectric composites, where static gradients enhance NP accumulation and alternating fields induce hyperthermia or mechanical stress to transiently open junctions [72]. In LNP-superparamagnetic iron oxide nanoparticles (SPION) hybrids, magnetic guidance has improved intra-tumoral distribution beyond angiogenic cores in rodent GBM models [72]. Emerging data indicate that synchronizing receptor-targeted LNPs with these physical modalities, particularly focused ultrasound or magnetic hyperthermia, extends parenchymal retention and cellular uptake, offering synergistic platforms for precise CNS gene therapy [69, 72].

## LNP Engineering for CNS Delivery

**Fig. 2:**
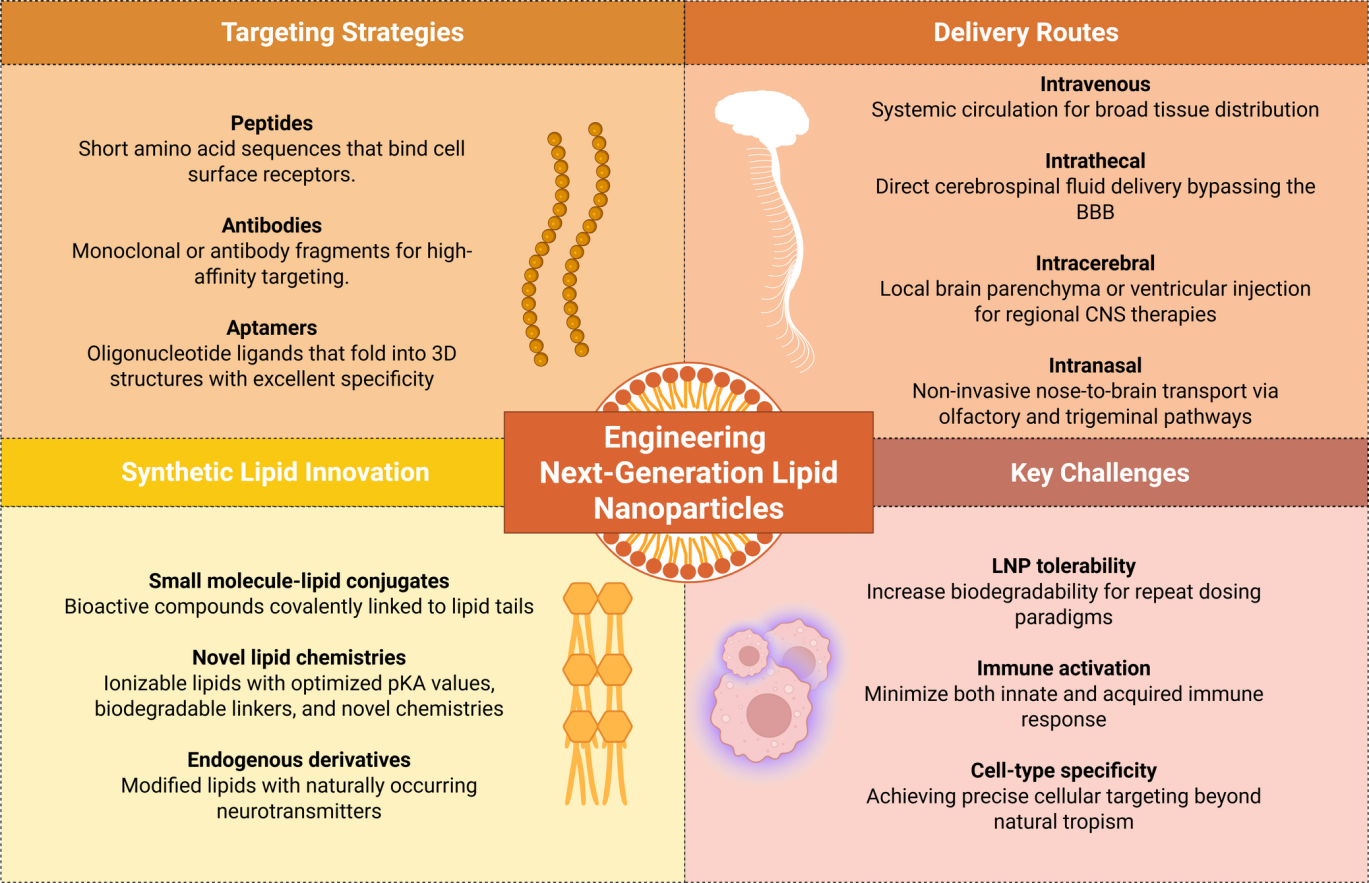
Considerations for the research and development of next-generation lipid nanoparticles (LNPs) for gene therapy applications (created with BioRender.com). *3D* three-dimensional, *BBB* blood–brain barrier, *CNS* central nervous system

An emerging area of innovation involves the development of synthetic lipid libraries providing an extensive repertoire of BBB-penetrating lipids (Fig. 2). This approach represents a paradigm shift from empirical formulation development to systematic structure–activity relationship studies that enable the rational design of brain-targeted delivery systems. Rational screening approaches have identified specialized lipids such as MK16 [73], TD5 [74], and NT1 lipidoids [75], which demonstrate dramatically improved brain accumulation compared to conventional US Food and Drug Administration-approved ionizable lipids such as DLin-MC3-DMA, used in first-generation mRNA vaccines such as Onpattro [76]. MK16, featuring a morpholine head group and optimized alkyl chain architecture, achieves up to 7.4-fold higher brain delivery compared with Food and Drug Administration-approved ionizable lipids following intravenous administration [73]. TD5 is a brain-targeting small molecule linked to an amino lipid that resulted in extensive green fluorescent protein expression in 29.6% of neurons and 38.1% of astrocytes across all major brain regions after intrathecal administration [74].

These novel lipids leverage precise chemical modifications to enhance lipid-membrane interactions, promote transcytosis across the BBB, and facilitate transfection in neuronal cells. Additional key design principles include optimization of pKa values to balance circulation stability with endosomal escape (typically targeting pKa values between 6.0 and 7.0), incorporation of biodegradable ester linkages to reduce accumulation toxicity, and fine-tuning of hydrophobic tail structures to optimize membrane fusion kinetics.

High-throughput screening approaches enable the simultaneous evaluation of thousands of lipid candidates, utilizing automated synthesis platforms, robotic handling systems, and high-content imaging to assess BBB penetration, cellular uptake, and toxicity profiles. Machine learning algorithms are increasingly employed to predict optimal lipid structures based on quantitative structure–activity relationships, potentially accelerating the identification of next-generation brain-targeted lipids [77].

The development of neurotransmitter-derived lipidoids represents a particularly elegant engineering strategy wherein lipid molecules mimic structural motifs of dopamine or serotonin, exploiting endogenous vesicular trafficking pathways to traverse the BBB [75]. These biomimetic lipids incorporate neurotransmitter-like head groups that potentially interact with cell surface monoamine transporters or vesicular monoamine transporters, providing a “Trojan horse” approach to BBB crossing. The neurotransmitter 1-lipidoid, which incorporates a dopamine-derived head group, demonstrates preferential accumulation in dopaminergic brain regions and has shown particular promise for delivering therapeutics in Parkinson’s disease models [75]. Similarly, linking a synthetic ligand for the 5-HT_3_ serotonin receptor, which is known to be transported into the CNS, to the LNP surface was shown to increase brain delivery of encapsulated mRNA [78].

In a notable example, Bian et al. [79] introduced a berberine-inspired ionizable lipid that enhanced nucleic acid therapeutic delivery across the BBB. By leveraging the structural features of berberine, known to interact with dopamine D_3_ receptor, and optimizing lipid architecture, this formulation achieved efficient brain targeting, endosomal escape, and potent gene expression in CNS tissues, further highlighting the potential of transporter-mediated uptake mechanisms. However, because the dopamine D3 receptor is predominantly expressed on neuronal and not endothelial cells, its contribution to direct RMT across the BBB is likely limited, suggesting that the observed brain delivery may primarily result from physicochemical mechanisms rather than true dopamine D3 receptor-driven transcytosis. These findings indicate that in addition to engineering novel lipids, the transporter-mediated mechanism represents a new avenue for BBB-crossing lipid design, potentially exploiting other endogenous transport systems for substrates such as glucose (glucose transporter 1), amino acids (LAT1), choline, and serotonin.

## Strategies for BBB-Targeted LNP Delivery

### Peptide-Based Targeting

The most extensively developed approach to BBB penetration of LNPs involves RMT, achieved through surface functionalization of LNPs with peptide-based targeting ligands. This mechanism exploits the natural vesicular transport pathways that evolved to facilitate the selective passage of essential nutrients and regulatory molecules across the BBB while maintaining barrier integrity [80]. Peptide-based targeting utilizes specialized sequences, such as T7 or angiopep-2, that specifically bind to receptors highly expressed on BECs, including TfR or LRP1 [81]. As with RMT (Fig. 1), the mechanism of peptide-mediated transcytosis involves several sequential steps: initial binding to surface receptors, triggering receptor clustering and membrane invagination, and subsequent formation of clathrin-coated vesicles that internalize the LNP-peptide complex. These vesicles then traffic across the endothelial cell cytoplasm, avoiding lysosomal degradation, and fuse with the basolateral membrane to release their cargo into the brain parenchyma [32]. Advanced dual-ligand or modular LNP designs can further enhance cell-type selectivity within the brain parenchyma, enabling preferential targeting of specific cell populations such as neurons, astrocytes, or microglia. These sophisticated systems incorporate primary targeting ligands for BBB crossing and the secondary neuronal-targeting ligand, Tet1 peptide, within a single peptide [82].

The use of cell-penetrating peptides (CPPs) has also been explored as a means to enhance CNS targeting with LNPs. In contrast to peptides that engage an RMT-capable receptor, CPPs are permeable to the cell surface membrane and interact with the plasma membrane in a receptor-independent manner [52]. For example, Cao et al. demonstrated that brain delivery of a brain-targeted LNP was enhanced by conjugation of the CPP TAT (derived from the HIV-1 protein TAT) on the surface of the LNP [78]. Similarly, the TAT peptide has been used in combination with transferrin to functionalize liposomes for CNS delivery and enhanced cellular uptake [83]. Notably, CPPs lack tissue specificity and must rely on additional BBB-permeable moieties to effect CNS targeting. However, their ability to enhance cellular uptake may represent a mechanism that can enhance post-BBB cellular uptake.

### Antibody-Based Targeting

Antibody-mediated targeting leverages the exceptional specificity of monoclonal antibodies or antibody fragments against BBB receptors, offering potentially superior selectivity compared with peptide-based approaches. The larger size and complex structure of antibodies provide multiple binding sites and extended serum half-lives, though they also present challenges related to immunogenicity and manufacturing costs [84].

Transferrin receptor antibodies conjugated to LNP surfaces have demonstrated superior BBB transcytosis and brain accumulation in preclinical models, facilitating more effective delivery of therapeutics to brain cells [85]. The transferrin receptor is particularly attractive as a BBB target because of its high expression on BECs, rapid internalization kinetics, and natural recycling pathway that avoids lysosomal degradation. Molecular studies have identified specific anti-TfR antibody clones that bind to distinct epitopes, enabling fine-tuning of transcytosis efficiency and brain penetration patterns [86].

Single-chain variable fragments offer the advantages of reduced size (approximately 25 kDa versus 150 kDa for full antibodies) while maintaining targeting efficacy [87]. The smaller size of single-chain variable fragments enables higher surface density decoration of NPs without steric hindrance, potentially improving avidity through multivalent interactions. Single domain antibodies have also emerged as highly promising targeting moieties for CNS-directed LNPs, offering distinct advantages over traditional antibody formats because of their small size (~ 12–15 kDa), enhanced stability, and reduced immunogenicity [88]. These compact antibodies can be effectively conjugated to LNP surfaces to facilitate BBB transcytosis.

A promising example is IGF1R5, a single domain antibody that targets the IGF1R, which is highly expressed on BECs [89]. IGF1R5 demonstrates sub-nanomolar binding affinity for IGF1R and mediates efficient transcytosis across both in vitro BBB models and in vivo systems, as evidenced by increased brain parenchymal uptake and CSF exposure. Importantly, IGF1R-targeting single domain antibodies exhibit cross-species reactivity and can be humanized without activating IGF1R signaling pathways, making them well suited for therapeutic applications. Incorporation of an IGF1R5 single domain antibody onto the LNP surface can help enable enhanced CNS penetration of RNA therapeutics.

It is noteworthy that antibody-mediated BBB transcytosis of biomolecules and NPs typically results in a small fraction of the injected dose (ID) being delivered to the CNS. For example, Johnsen et al. [90] reported that < 0.5% of the ID anti-TfR-labeled gold NPs were detected in the brain. However, certain reports have indicated that delivery of up to 4% of the ID was achieved using TfR-targeted NPs. This level of delivery appears to be generally consistent with reports of TfR-targeted antibodies, where 3% of the ID was observed [91]. There are many factors that affect an antibody’s capacity to mediate RMT, including affinity, avidity, valency, and pH sensitivity [5]. While efforts to optimize antibody characteristics and prolong the serum half-life can have dramatic effects on the delivery of biotherapeutics to the CNS [92], the inherent capacity of RMT may represent an upper limit to CNS delivery based on this mechanism. If the RMT mechanism is a limiting factor, then the therapeutic capacity of LNPs should be superior to that of a fusion protein. More importantly, it is often observed that peripheral tissues, such as the liver and spleen, accumulate the majority of the ID [90]. Thus, further optimization of RMT-mediated CNS delivery could be achieved by detargeting peripheral tissues, thereby reducing the peripheral sink effect. Recently, Gentry et al. [93] screened lipid libraries to identify an LNP formulation that detargets the liver, thereby altering the tropism toward spleen delivery. This breakthrough represents the possibility for the identification of an LNP formulation that detargets peripheral tissues and becomes increasingly reliant on the antibody–target interactions for tissue uptake. This could be expected to increase the percentage of the ID delivered to the CNS via RMT.

### Aptamer-Based Targeting

Aptamers represent an emerging class of targeting moieties consisting of short single-stranded oligonucleotides (typically 20–100 nucleotides) that fold into unique three-dimensional structures capable of binding specific protein targets with high affinity and specificity [94]. These nucleic acid-based ligands are selected through systematic evolution of ligands by exponential enrichment (SELEX), a process that screens libraries containing up to 10^15 unique sequences to identify optimal binders. These molecules represent a compelling alternative to both peptides and antibodies, maintaining high target specificity (dissociation constants often in the picomolar to nanomolar range) while presenting minimal immunogenicity owing to their nucleic acid composition and a lack of protein epitopes. Unlike antibodies, aptamers can be chemically synthesized with high batch-to-batch consistency for a lower-cost scale-up and can be modified with functional groups for easy conjugation to LNPs.

Aptamer-functionalized NPs have been engineered to target BBB receptors [95]. In this example, a targeting strategy involving LNPs functionalized with anti-platelet-derived growth factor receptor-beta aptamers could both facilitate RMT across the BBB and selectively accumulate in glioblastoma in preclinical models. The small size of aptamers compared to antibodies (molecular weight typically 5–30 kDa vs 150 kDa for antibodies) also enables denser NP surface modification without steric hindrance [96].

## Alternative Administration Routes to Bypass the BBB

### Intranasal Delivery

Intranasal delivery of LNPs exploits direct neuronal pathways through the olfactory and trigeminal nerves to circumvent the BBB entirely, providing rapid brain access with reduced systemic exposure [97]. Although limited examples exist for ionizable cationic-based LNPs, there is some evidence that cationic liposomal mRNA NPs can be administered intranasally for brain transfection in mice [98]. A single intranasal dose (3 mg/kg) yielded 12-fold higher brain expression in the cortex versus naked mRNA, with dose-dependent luciferase activity distributed across the cortex, striatum, and midbrain and minimal systemic exposure [98]. Although intranasal LNP delivery may enable direct brain access via olfactory and trigeminal pathways while avoiding systemic exposure, it currently faces major limitations, including small dosing volume, mucociliary clearance, variable nasal anatomy, low penetration efficiency with non-viral carriers, potential local toxicity, poor scalability from rodents to humans, and a lack of robust data for ionizable formulations.

### Intracranial Injection

Intracranial injection allows direct delivery of LNPs into the brain parenchyma, completely bypassing the BBB and enabling high local concentrations with minimal systemic exposure. This approach is particularly valuable for treating localized brain pathologies, such as glioma. Studies using MC3-based LNPs demonstrated efficient delivery of mRNA, including Cre recombinase mRNA and Cas9 mRNA/single-guide RNA, to various mouse brain regions, including the striatum and hippocampus. Transfection efficiencies reached approximately 50% in striatal and hippocampal neurons, astrocytes, and microglia following direct intracranial injection [99]. Generally, intraparenchymal injections achieve high local concentrations, but with limited diffusion. The limited penetration results from the dense extracellular matrix of brain tissue, which restricts NP diffusion, and the lack of convective flow in brain parenchyma.

### Intracerebroventricular Administration

Intracerebroventricular (ICV) injection represents a strategy for delivering LNP-encapsulated RNA therapeutics directly into the CNS, bypassing the BBB and enabling more widespread distribution via CSF [100]. The ventricular system provides a natural distribution network throughout the brain, with CSF flow patterns that facilitate drug dispersion to periventricular and deeper brain structures.

In a pivotal study, LNPs formulated with siRNA targeting CAG repeat expansions were administered via ICV injection in a mouse model of polyglutamine disorders. This approach achieved selective knockdown of mutant mRNA transcripts across key brain regions, including the cortex and striatum, with minimal impact on wild-type alleles. The treatment significantly reduced polyglutamine-expanded protein levels, demonstrating both the specificity and efficacy of ICV-delivered LNPs in silencing disease-causing genes [101]. Although ICV delivery of LNPs allows for direct access to the CNS and broad cerebrospinal fluid distribution, it should be noted that ICV injections can be associated with significant complications, including infectious and non-infectious complications in up to 27% and 33% of patients, respectively [102].

### Intrathecal Delivery

Intrathecal delivery places LNPs into the CSF within the spinal canal, resulting in widespread CNS distribution through CSF circulation. This route offers several advantages over ICV injection, including easier surgical access, reduced risk of brain injury, and potentially more uniform distribution throughout the CNS [103].

Recent research indicates that intrathecal LNP administration can achieve substantial gene transfer, protein expression, and functional gene editing across multiple brain regions [74]. Distribution studies demonstrate that intrathecally injected LNPs reach brain tissue within 30–60 minutes, with peak concentrations occurring 2–4 hours post-injection. This route harnesses CNS fluid dynamics to distribute NPs while circumventing systemic clearance and achieving higher localized CNS exposure compared with intravenous injection [104].

Clinical development efforts utilizing intrathecal LNP delivery for rare CNS diseases and pediatric applications where broad brain exposure is essential are currently underway [104]. Spinal muscular atrophy treatment with ASOs has validated the clinical feasibility of intrathecal nucleic acid delivery, and several gene therapy trials are exploring intrathecal administration of viral vectors [105]. The translation of LNP technology to intrathecal delivery builds upon this established clinical precedent while potentially offering advantages in cargo versatility and repeat dosing.

## Clinical Translation and Safety Profile

### Therapeutic Efficacy

Modern LNP platforms for CNS delivery have demonstrated promising in vivo performance across multiple validated preclinical and emerging clinical contexts, characterized by effective therapeutic outcomes and favorable safety profiles. The transition from proof-of-concept studies to therapeutic validation represents a critical milestone in the development of CNS nanomedicine. In preclinical disease models including AD, gliomas, and neuroinflammation, LNP formulations carrying mRNA, siRNA, or protein therapeutics have achieved marked improvements in behavioral metrics and significant attenuation of pathological hallmarks, including amyloid-beta aggregation, tumor burden, and inflammatory cytokine levels [106–108].

### Enhanced Tolerability

The tolerability of next-generation LNP platforms has improved substantially through structural and compositional refinements, such as biodegradability. Early LNP formulations often caused dose-limiting toxicities, including complement activation, cytokine release syndrome, hepatotoxicity, and immunogenicity, limiting their therapeutic window and repeat dosing potential [109]. Incorporating biomimetic surface coatings and replacing traditional polyethylene glycol with smart polyethylene glycol alternatives or stealth ligands minimizes complement activation, reduces immunogenicity risks, decreases cytokine release, and attenuates hepatic accumulation and toxicity [110, 111]. These improvements are particularly important for long-term administration scenarios where repeat dosing regimens may be necessary, such as in neurodegenerative and complex genetic disorders. Chronic toxicology studies in non-human primates have demonstrated that optimized LNP formulations can be administered weekly without cumulative toxicity, dose-limiting adverse effects, or significant immune sensitization [112].

## Clinical Trials Involving LNP Delivery Systems

Lipid nanoparticle-mediated delivery systems have now advanced from conceptual platforms to clinically validated modalities spanning RNA interference, in vivo gene editing, and emerging epigenome-targeting therapies, with several key programs defining the translational landscape [113–118]. The first major milestone was patisiran (Onpattro^®^; Alnylam), an intravenously administered LNP-formulated siRNA that targets transthyretin (TTR) in hepatocytes for the treatment of hereditary transthyretin-mediated amyloidosis with polyneuropathy, which established the feasibility of repeated systemic LNP dosing in humans [113]. Building on this liver-directed paradigm, in vivo CRISPR-Cas9 gene editing entered the clinic with NTLA-2001 (Intellia/Regeneron), an LNP-encapsulated CRISPR-Cas9 mRNA and guide RNA combination designed for single-dose TTR knockout in hereditary transthyretin-mediated amyloidosis, where first-in-human data demonstrated substantial and durable serum TTR reduction and provided proof-of-concept for permanent gene editing via LNPs [114].

Beyond TTR, a new generation of cardiovascular gene editing candidates use LNPs to deliver base-editing or CRISPR-Cas9 components to hepatocytes to durably reduce atherogenic lipoproteins [115]. Verve 101, an in vivo base-editing therapy targeting PCSK9, is being evaluated for heterozygous familial hypercholesterolemia and high atherosclerotic risk, and early clinical summaries emphasize LNP-mediated liver delivery as a central feature [116, 119]. Additional programs such as CTX310 (ANGPTL3 editing) similarly rely on systemic ionizable LNPs to enable one-time or infrequently dosed interventions for cardiovascular risk reduction, reflecting a broader shift toward LNP-delivered genome editors in common diseases [117]. In parallel, epigenome editing approaches extend the LNP delivery to CRISPR-based transcriptional modulation, illustrating how LNPs can support not only gene knockout or correction but also durable programmable regulation of pathogenic loci in the liver [118].

Clinical trials are also underway investigating LNP delivery for mRNA replacement therapy. ARCT-810 (LUNAR©-OTC) is a systemically (intravenous infusion) delivered LNP-mRNA that aims to treat ornithine transcarbamylase deficiency by supplying an mRNA encoding a functional enzyme to the liver [120]. Similarly, ARCT-032 (LUNAR©-CF) is an LNP-mRNA formulation that is locally administered to the lung, via aerosolization, to deliver functional cystic fibrosis transmembrane conductance regulator mRNA to the lung.

These efforts underscore how incremental advances in LNP design and cargo technology translate directly into improved transfection, safety, and manufacturability in the clinical setting. Together, these approved products and early-stage trials define a rapidly evolving translational landscape in which LNPs function as a modular delivery chassis for siRNA, mRNA, and genome or epigenome editors, and they provide a clinically grounded framework for new LNP designs.

## Current Challenges and Limitations

Despite significant progress, several challenges persist in LNP-mediated CNS delivery that must be addressed to achieve the full therapeutic potential of this technology. Precise regional and cell-type targeting within the heterogeneous CNS environment remains a challenge, with existing approaches falling short of reliably targeting discrete cell populations such as hippocampal neurons, dopaminergic cells, or microglia with high specificity [121]. The challenge of cellular heterogeneity is compounded by regional differences in BBB properties, vascular density, and extracellular matrix composition [122]. For example, the BBB in the hippocampus exhibits different transporter expression profiles compared with cortical regions, while the BBB in pathological conditions such as AD or multiple sclerosis shows altered permeability and receptor expression patterns [123]. These variations necessitate disease-specific and region-specific optimization of LNP formulations [124].

## Gene Therapy for the CNS

Central nervous system disorders are frequently caused by monogenic or polygenic mutations that lead to dysfunctional proteins or dysregulated gene expression. Gene therapy offers the potential to directly address the underlying genetic cause of these diseases. This is especially critical where early or timely intervention could prevent irreversible damage. As the symptoms of genetic diseases with CNS pathology, such as lysosomal storage disorders, are typically evident during development, they require early intervention to limit or prevent developmental abnormalities. Moreover, timely intervention in late-onset diseases, such as AD or PD, could prevent lasting damage and enable healthy aging.

The introduction of gene regulatory and editing tools and technologies has enabled researchers to design targeted therapies to treat genetic diseases and conditions. Many tools have been developed for gene editing, including zinc-finger nucleases, transcription activator-like effector nucleases, and CRISPR. While zinc-finger nucleases and transcription activator-like effector nucleases are valid gene editing tools, the comparative ease and versatility of CRISPR have accelerated gene therapy research [125]. Recent clinical successes include the use of CRISPR-Cas9 to correct CEP290 gene mutations that cause childhood blindness in Leber congenital amaurosis (LCA10), and the disruption of the BCL11A enhancer in sickle cell disease [126, 127]. This highlights the transformative potential of CRISPR-Cas gene therapy and precision medicine to treat previously incurable conditions and emphasizes the need to explore implementation strategies for CNS disorders. As described above, the BBB is a significant obstacle that increases the complexity of implementing gene therapy strategies targeting the CNS. With the recent advances in CNS-targeted LNP technology, the feasibility of gene therapies based on CRISPR-Cas systems and RNA therapeutics has increased, with effective treatment modalities for rare neurological and neurodegenerative diseases on the horizon (Table 1).

**Table 1:** Central nervous system disorders related to genetic dysregulation and emerging therapeutic strategies

Disease	Gene(s) regulatory pathways involved	Type of dysregulation	Therapeutic interventions	References
Alzheimer’s disease	*APP, PSEN1/2, APOE, MAPT*	Mutations, epigenetic changes, misfolded proteins	CRISPRa/i for neuroprotection, siRNA for tau silencing, AAV-based gene therapy	[128–130]
Parkinson’s disease	*LRRK2, SNCA, PINK1, PARK7*	Gain/loss-of-function mutations	siRNA/ASOs against *SNCA*, CRISPR knock-in/out, base editing	[131–133]
Huntington’s disease	*HTT* (CAG trinucleotide repeat)	Toxic gain-of-function	CRISPR/Cas9, knockdown, base editing, CRISPR/CasRx, ASO therapy	[134–137]
Rett syndrome	*MECP2*	Loss-of-function mutation	Base editing, CRISPR gene replacement	[138–140]
Glioblastoma	Not fully described but prognostic markers include: *NOTCH1, MYC, TOP2A, CD44, PTPRC, SSRP1, CDK4*	Oncogenic mutations, transcriptional dysregulation	CRISPRa/i, epigenetic modifiers	[141–144]
Leukodystrophies	*GALC, ARSA, ABCD1*	Enzyme deficiency due to mutations	ASO, CRISPR/Cas9	[145, 146]
Spinal muscular atrophy	*SMN1* deletion, *SMN2* splicing defects	Loss of functional protein	ASO (e.g., nusinersen), base editing	[147–149]
LSDs	Depending on which LSD various targets can be used, GLA, GBA1, and SMPD1	Enzyme deficiencies	CRISPR/Cas9	[150–153]

*LSDs* lysosomal storage disorders *ASO* antisense oligonucleotide, *CRISPR* clustered regularly interspaced short palindromic repeats, *siRNA* small interfering RNA, *AAV* adeno-associated virus

## Application of CRISPR Technologies

**Fig. 3:**
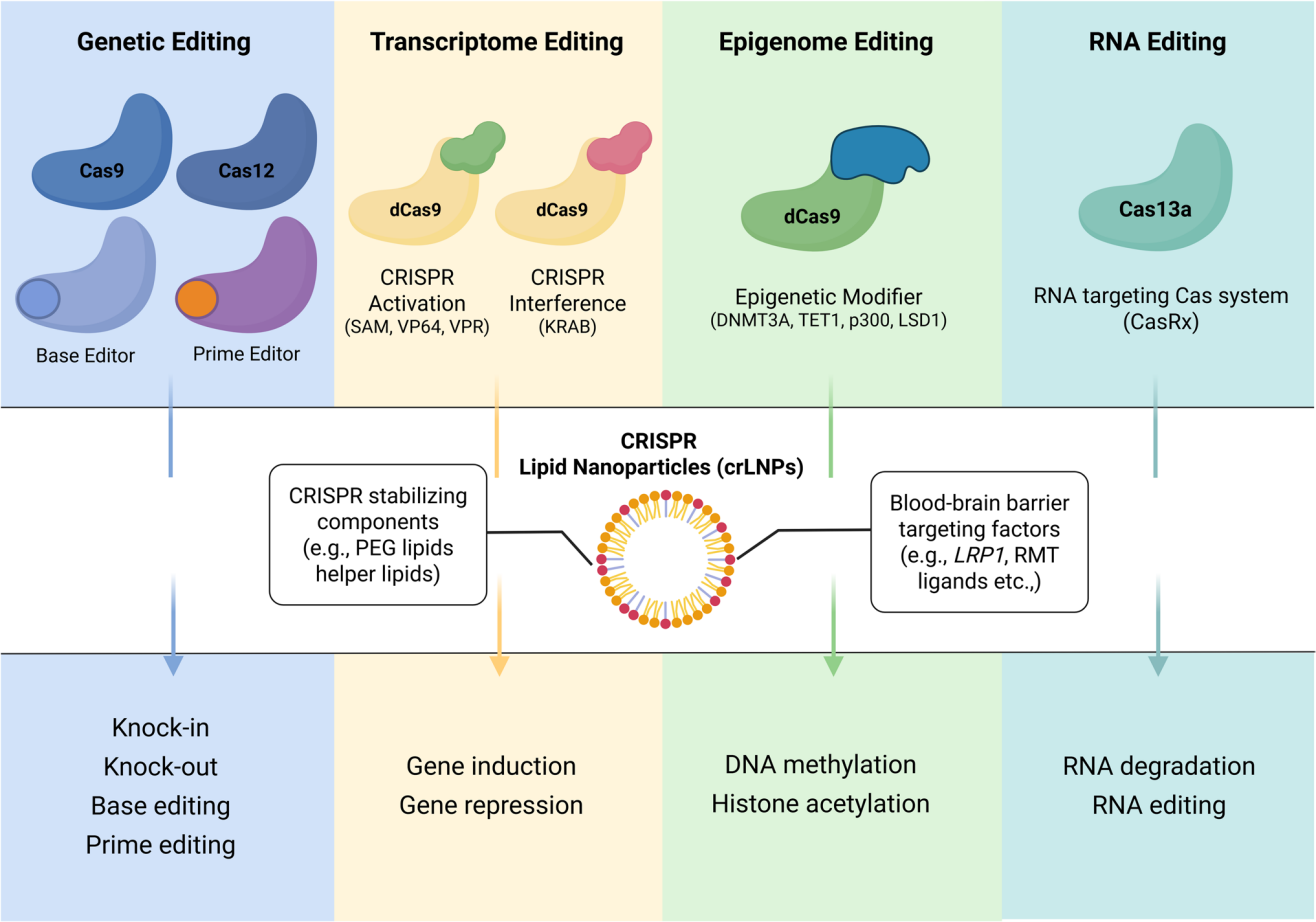
Clustered regularly interspaced short palindromic repeats (CRISPR)-Cas modalities delivered by brain-targeted CRISPR lipid nanoparticles. DNA targeting editors, including Cas9, Cas12, base editors, and prime editors, enable direct genome modification. Transcriptome modulation is achieved with catalytically inactive dCas9 fused to transcriptional effectors, while epigenome editing employs dCas9 fused to DNMT3A or TET1 to induce DNA methylation or histone modification. CasRx facilitates RNA editing by degrading or modifying transcripts, thereby altering protein expression (created with BioRender.com). *PEG* polyethylene glycol, *RMT* receptor-mediated transcytosis

CRISPR-Cas systems are quickly expanding and evolving technologies for genetic engineering that are continually being adapted for novel applications. CRISPR systems have been developed for diverse applications, including gene knockout, knock-in, sequence editing, transcriptional modulation, and epigenetic editing (Fig. 3). However, gene therapy based on AAV vectors is limited by several factors, including restricted payload capacity, immunogenicity, inefficient CNS penetration, and patient reluctance. In contrast, LNPs are a promising alternative to deliver gene therapies because of their higher nucleic acid and/or protein payload capacity, which enables the use of larger next-generation CRISPR technologies (such as prime editors and PASTE).

### CRISPR Knockout, Knock-in, Knockdown, and Base/Prime Editing

The most common, well-studied CRISPR technologies employ Cas9 or Cas12a nucleases [154, 155] to create a targeted double-strand break, resulting in either a gene knock-out by non-homologous end joining or gene insertion by homology-directed repair [156]. This enables the irreversible silencing of toxic alleles/genes or the insertion or correction of nucleotides. More recent novel experimental approaches, such as PASTE (programmable addition via site-specific targeting elements), can be used to enhance site-specific integration efficiency without the reliance on homology-directed repair [157].

CRISPR knockout and knock-in technologies are increasingly being used for gene therapy. For example, Rett syndrome is a severe X-linked neurodevelopmental disorder caused by loss-of-function mutations in the *MECP2* gene [158, 159]. Several studies have investigated CRISPR-based approaches to correct *MECP2* mutations in human cell lines and induced pluripotent stem cell lines [160, 161]. Notably, Cho et al. demonstrated NP-assisted CRISPR-Cas9 delivery into iPSC-NPCs, successfully restoring *MECP2* function by homology-directed repair-mediated insertion of a corrective gene sequence and generating neurons that phenotypically reflect wild-type neurons [139]. In another example, HD is a dominant inherited neurodegenerative disorder caused by CAG trinucleotide repeats in the *HTT* gene that result in gradual CNS dysfunction [162, 163]. CRISPR-Cas9-mediated gene knockout was performed to disrupt mutant *HTT* alleles in mice using AAV delivery [164], which resulted in reduced motor deficits and extended survival.

Another major advancement in the CRISPR-Cas system is the development of base and prime editing technology. Base editing and prime editing enable precise single or short nucleotide edits without introducing double-stranded breaks [165]. Cytosine and adenine editors enable conversion of point mutations (C to T or A to G) while prime editors expand the range of possible edits up to 12 types of substitutions [166, 167]. These technologies can address monogenic neurological diseases where single-nucleotide mutations result in disease phenotypes. For example, alternating hemiplegia of childhood is a rare neurodevelopmental disorder associated with mutations in the *ATP1A3* gene, causing motor and cognitive impairment [168]. A recent study employed prime and base editing technology in both human cells and alternating hemiplegia of childhood mouse (*ATP1A3*) models to reverse its associated pathological phenotypes [169]. They were able to demonstrate that in vivo delivery of a prime editor targeting the *ATP1A3* gene was capable of correcting five different mutations, ameliorating both motor and cognitive defects, and drastically increasing lifespan. This represents a compelling example of a single-dose gene therapeutic that can be used as a template for other rare neurological diseases.

In addition to DNA-targeting systems, CRISPR-Cas13 (also known as CasRx) has been recently adapted for post-transcriptional gene modulation through mRNA knockdown [170]. Unlike Cas9 or Cas12, which cleave DNA, CasRx binds to and degrades RNA molecules, offering an alternative avenue for reversible gene suppression. A recent study utilized CasRx to knock down mutant *HTT* mRNA in HD mice (HD140Q-KI) and HD pig (HD-KI) models to address their associated pathology [135]. Consequently, they were able to ameliorate gliosis in mice and delay neurodegeneration in pigs, underscoring the therapeutic potential of RNA-targeting CRISPR tools for treating these disorders.

### CRISPR Transcriptional Regulation and Epigenome Editing

CRISPR-based transcriptional regulation systems, CRISPR activation and CRISPR interference, use a catalytically dead Cas enzyme (such as dCas9 or dCas12a) fused with transcriptional activators or repressors to modulate gene expression (Fig. 3) [171, 172]. These systems do not cut DNA and allow for reversible modulation of gene expression, which is especially valuable in CNS disorders where dynamic regulation of gene expression represents the safest treatment modality.

In a demonstration of the utility of CRISPR activation in AD, Park et al. employed a dCas9-activator nanocomplex in mice to induce the expression of *ADAM10*, a member of the α-secretase family that cleaves APP, to decrease dementia-causing plaques in the brain [173]. Similarly, Colasante et al. demonstrated the use of CRISPR activation to induce *Snca1* gene expression to rescue inhibitory interneuron excitability and halt seizures in Dravet syndrome [174]. These proof-of-concept studies demonstrate the potential of regulating endogenous gene expression as a strategy to correct pathologies arising from conditions associated with aberrant gene expression.

CRISPR-Cas systems have also been developed to modify epigenetic signatures by manipulating histone modifications, DNA methylation, chromatin accessibility, and three-dimensional genome organization [175]. These epigenome-editing systems typically employ a dCas9 fused to effector domains including DNA methyltransferase (e.g., DNMT3A), demethylases (e.g., TET1, LSD1), or histone acetyltransferases (e.g., p300) [176]. Researchers have developed tools capable of repressing gene expression with DNA methylators such as dCas9-DNMT3A [177] and also inducing gene expression through demethylators such as dCas9-TET1 [178, 179]. Patients with AD exhibit increased amyloid precursor protein (*APP*) expression, which is thought to trigger dementia-causing plaque formation in the brain [180]. Park et al. aimed to repress APP expression by hypermethylating the *APP* promoter in *APP-KI* mice with dCas9-DNMT3A [181]. This intervention reduced overall mRNA expression, decreased neuronal cell death, and attenuated cognitive and behavioral impairments. Similarly, overexpression of α‐synuclein in PD is linked to elevated H3K4me3 enrichment at the SNCA promoter [182–184]. Guhathakurta et al. developed dCas9 Suntag-JARID1A to remove H3K4me3 in the α‐synuclein promoter and successfully decreased its expression in both SH-SY5Y cells and iPSC-derived dopaminergic neurons [184]. Similarly, Liu et al. demonstrated that targeting the CGG repeat expansion in the FMR1 promoter with dCas9-Tet1 reduced methylation of the promoter and restored FMR1 gene expression in FXS-induced pluripotent stem cell lines [185]. These examples showcase how targeted epigenetic editing can correct disease-associated dysregulation without altering genomic sequences.

### LNP Encapsulation of CRISPR Components

Major challenges to the clinical implementation of CRISPR-based therapeutics are the efficient and precise delivery of large molecular payloads across the BBB and nuclear localization following uptake by the target cell. Lipid nanoparticles are well suited to solving these challenges as they are capable of encapsulating large complex CRISPR systems comprised of Cas enzymes, single-guide RNAs, and effector domains. The size and physiochemical properties of ribonucleoprotein complexes add to the challenge of developing LNPs for CRISPR-Cas systems. This is particularly true for Cas enzymes that have been functionalized with additional enzymatic domains, such as the PASTE system, which is a 307-kDa fusion of dCas9, a reverse transcriptase and a serine integrase [157]. Moreover, Cas endonucleases are cationic in nature and pose a barrier to stable encapsulation [186]. Intracellular delivery adds another layer of complexity, requiring that the packaged components escape the endosome and undergo nuclear localization for functional enzymatic activity [125, 187].

Recent developments in CRISPR-loaded LNPs have demonstrated efficient gene editing in cell and animal models [188–191]. These included optimizing ionizable lipids, endosomal release, and incorporation of helper and PEG lipids to mediate distribution and reduce toxicity [188]. Despite these successes, CRISPR-loaded LNPs are designed to target peripheral tissues. A recent study described the development of a BBB-penetrable nanocapsule containing a Cas9-single-guide-RNA complex within a glutathione-polymer sensitive shell with ligands targeting a receptor highly expressed in BECs, such as LRP1 [192]. Zou et al. showed that these CRISPR-loaded LNP effectively downregulated *PLK1* expression in tumor cells and doubled the survival rate in a glioblastoma mouse model [192]. This work demonstrates the potential of BBB-specific RMT ligands and antibodies to enable the delivery of large complex gene editors based on CRISPR-Cas technology to address neurological and neurodegenerative disorders.

## Optimizing the Delivery of DNA-Based and RNA-Based Therapies for CNS Disorders

RNA and DNA therapeutics are a modality that can be used for a wide variety of diseases, including neurological and neurodegenerative disorders. Unlike genome-editing tools, these strategies offer transient and or reversible modulation of gene expression [193]. These therapies can be engineered to silence or degrade pathogenic transcripts, upregulate gene expression, encode therapeutic proteins, or modulate disease-associated pathways [193, 194]. This makes nucleic acid-based therapeutics particularly attractive for neurological and neurodegenerative diseases and can be rapidly tailored to both monogenic and polygenic CNS disorders.

### Regulatory RNA Modalities for CNS Therapeutic Intervention

Key classes of regulatory RNA therapeutics include ASOs, siRNAs, and microRNAs (miRNAs), which enable sequence-specific modulation of genes through post-transcriptional mechanisms. Among these, ASOs are short, single-stranded (~12–30 nucleotides) nucleic acid molecules that bind to RNA targets to modulate gene expression by affecting RNA processing or degradation [195]. Antisense oligonucleotides rely on RNAse H-mediated degradation of mRNAs to silence the expression of targeted genes [196, 197]. Several ASO-based drugs have received regulatory approval as gene therapy for neurological disorders such as Duchenne muscular dystrophy, spinal muscular atrophy, hereditary transthyretin-mediated amyloidosis, and amyotrophic lateral sclerosis [198]. Despite their success, the delivery of ASOs to the brain remains a challenge, often relying on invasive intrathecal injections into the CSF with limited tissue distribution [199]. In contrast, siRNAs are double-stranded RNA molecules (~ 20–25 base pairs) that trigger mRNA degradation through the RNA-induced silencing complex [200]. Numerous siRNAs are in the clinical stages of development, such as Mivelsiran and BIIB080 targeting APP mRNA for AD [201]. Other siRNA therapeutics have progressed to phase II trials [202, 203]; however, similar to ASOs, siRNA therapies typically rely on intrathecal delivery. MicroRNAs are small non-coding RNA molecules that play an implicit role in post-transcriptional gene regulation by binding to mRNA targets [204]. Therapeutic strategies using miRNAs include using synthetic miRNA mimics to restore downregulated miRNAs or antagomirs (anti-miRNAs) to inhibit overactive miRNAs [204, 205]. Combinatorial miRNA-based therapies are in development for conditions such as AD with the potential to simultaneously target various components of pathogenic pathways [201]. Despite the distinct molecular mechanisms of ASOs, siRNAs, and miRNAs, they converge functionally as precise tools for post-transcriptional gene regulation, offering an alternative strategy for transient and tunable modulation without permanently altering the genome. Collectively, regulatory RNA modalities show substantial promise, evidenced by the growing pipeline of clinically validated therapeutic interventions. This is mainly owing to their unique properties such as target specificity, reversibility, and dosing flexibility. However, broader translation to the CNS context remains limited by invasive routes of administration, RNA stability and bioavailability at the site of action, as well as challenges in achieving widespread parenchymal distribution across the BBB.

## mRNA Therapeutics for the CNS: Programmable, Transient, and BBB-Targeted Delivery

Messenger RNA therapeutics have gained traction in recent years owing to the success of the mRNA-based coronavirus disease 2019 vaccine, which demonstrated that synthetic mRNA can be safely delivered to humans [206]. This success, combined with the accompanying LNP development and validation, has invigorated research into mRNA therapeutics for a broad spectrum of diseases. Lipid nanoparticles protect mRNA from degradation, facilitate cellular uptake, and provide a versatile delivery [73, 207] platform through the BBB, making mRNA now an attractive solution for transient protein expression. Moreover, recent efforts in chemical modifications to mRNA, such as nucleoside analogs, optimized untranslated regions, and poly-A tail engineering, enhance RNA stability and reduce innate immune activation, thereby improving their translational efficiency [208, 209]. Collectively, these properties enable a tunable and transient protein production strategy, which can be an advantage when precise temporal control is required. The therapeutic landscape for mRNA therapeutics in the CNS context was broadly explored for immunological and functional protein replacement strategies. For example, LNP-mRNA platforms achieved human trials (NCT04573140) for personalized immunotherapies for high-grade gliomas and adult glioblastomas by stimulating an anti-tumor response [210, 211]. Moreover, preclinical models for PD and AD leveraged mRNA to achieve transient and high expression of neurotrophic factors, such as brain-derived neurotrophic factor, and anti-inflammatory cytokines [129, 212–214] in the brain. This can be particularly advantageous in the CNS as it can avoid overexpression toxicity and immune activation associated with conventional viral-mediated gene delivery approaches.

Beyond protein replacement therapies, mRNA platforms also support programmability with switch and biosensor technologies leveraging regulatory RNAs [215]. Recent advances in miRNA-responsive RNA switch technology exploit endogenous miRNA expression profiles to selectively regulate mRNA translation. For example, the Hirohide laboratory developed programmable mRNA circuits incorporating miRNA recognition elements flanking a reporter gene to conditionally activate and repress translation based on miRNA recognition [216]. Incorporating miRNA sensors as ON/OFF switches resulted in tunable cell type-specific protein expression, minimized protein leakiness, and suppression of off-target expression. Importantly, this RNA platform was also delivered in vivo, achieving tissue-specific activation of protein production in mouse liver and muscle. In the CNS, where cell-type heterogeneity and specificity are challenges for targeted delivery, solutions such as these pave the way for precise and temporally regulated RNA-based therapeutics targeting specific cell types past the BBB. Integration of miRNA-responsive switches with LNP delivery systems may also further improve translational feasibility by coupling non-viral delivery with endogenous regulatory control.

Despite these advances, mRNA therapeutics are challenged by rapid degradation and limited stability when administered conventionally [217]. Lipid nanoparticle technology has partially addressed these limitations by enhancing bioavailability, reducing immunogenicity, and protecting mRNA payloads from degradation. However, further optimization of LNP-RNA platforms is required to improve their translational performance in clinical settings. Continued innovation in the mRNA and regulatory RNA design alongside LNP engineering expands the scope of CNS-targeted therapies by enabling tunable, transient, and safe interventions, including vaccine and protein replacement strategies for a broad range of neurological disorders.

## DNA-Based Gene Therapies for the CNS: Sustained Expression and Next-Generation Vectors

While RNA-based therapies emphasize tunability and safety, DNA-based gene delivery remains attractive for applications that require sustained transgene expression in the CNS. Conventional plasmid DNA vectors (3–10 kb) support episomal gene expression lasting days to weeks in dividing cells and weeks to months in non-dividing tissues, with well-established scalability and low manufacturing costs [218, 219]. Expression durability is highly tissue dependent; skeletal muscle can sustain expression for over a year, whereas hepatic expression often declines within days because of promoter inactivation and immune clearance [220]. Plasmid DNA has been evaluated as a treatment for chronic neurodegenerative disorders where long-term therapeutic expression is desired. A prominent example of plasmid DNA-based CNS therapy is the delivery of the protective apolipoprotein E2 (*APOEε2*) isoform to mitigate neurodegeneration associated with AD. *APOEε* genotypes are central to AD risk, where homozygous *APOEε4* carriers develop increased risk for disease onset [221–223]. In contrast, *APOEε2* is protective and can offset the deleterious effects of *APOEε4* [224]. Early preclinical studies using intracerebroventricular lentiviral vector administration in mice demonstrated reduced hippocampal plaque formation [225]. Subsequent studies in larger animal models evaluated intraparenchymal, intracisternal, and intraventricular delivery and revealed that intracisternal administration offered the best balance of efficacy and limited invasiveness [222]. However, these approaches were still limited by sub-optimal distribution, immunogenicity, and invasiveness. Emerging non-viral strategies such as plasmid DNA packaged in liposomes and LNPs tagged with transferrin and penetratin were explored as BBB penetrant alternatives [226, 227]. These approaches achieved successful delivery and transgene expression in mice, highlighting the potential of a less-invasive gene-supplementation strategy in the CNS.

The major limitations faced by plasmid DNA include poor nuclear import of large double-stranded DNA in quiescent cells, CpG-mediated activation of innate immune pathways such as cGAS-STING, and transcriptional silencing caused by bacterial sequences [220]. Although LNPs enhance cellular uptake and endosomal escape, they do not intrinsically overcome nuclear entry barriers and can provoke dose-limiting inflammation. To overcome these barriers, next-generation plasmid technologies are being developed to improve the feasibility of DNA-based gene therapy. These advanced DNA vector formats, including mini-string DNA, closed single-stranded DNA, minicircle DNA, and nanoplasmid DNA, have been developed and are increasingly paired with LNP delivery systems to improve nuclear access, reduce immunogenicity, and prolong expression duration [218, 228–234]. Mini-string DNA and closed single-stranded DNA are substantially smaller vectors that lack bacterial backbone elements, thereby improving nuclear delivery and reducing immunogenicity [231]. Mini-string DNA consists of compact (2-4 kb) backbone-free constructs that exhibit two- to ten-fold higher transgene expression than conventional plasmids across multiple cell types, with minimal innate immune activation [228]. Closed single-stranded DNA is a closed single-stranded circular format produced enzymatically that has demonstrated high efficiency gene insertion, particularly in hematopoietic stem and progenitor cells, with reduced toxicity compared with double-stranded DNA and greater stability than RNA [229, 230]. Despite their favorable biological profiles, both formats face challenges related to production complexity [228–230].

Minicircle DNA vectors, generated by site-specific recombination to remove the bacterial backbone sequences, typically range from 2-6 kb and exhibit markedly enhanced nuclear import and transcriptional persistence [235]. Minicircles achieve 10- to 1000-fold higher expression than parental plasmids and support sustained expression for weeks in dividing cells and months in non-dividing tissues following an initial decline [236]. Although early production methods were labor intensive and low yielding, advances in producer strain engineering have significantly improved scalability, enabling broader translational use [236]. Lipid nanoparticle-based delivery strategies for minicircle DNA, including targeted formulations, are now under active development.

Nanoplasmid DNA vectors represent an intermediate strategy that preserves plasmid manufacturability while minimizing immunogenicity. These vectors employ ultra-short bacterial backbones (≤ 500 bp) and antibiotic-free RNA-based selection systems, reducing CpG content and transgene silencing [231]. Nanoplasmids maintain high fermentation yields, approaching 2 g/L, and have demonstrated favorable safety and efficacy in clinical studies, with improved expression compared with conventional plasmids [231, 232]. Notably, robust nanoplasmid delivery and expression have been achieved in vitro and in vivo when paired with LNP systems [237, 238].

Across all DNA vector formats, innate immune sensing of cytosolic DNA remains a key limitation for LNP-mediated delivery. Activation of the cGAS-STING pathway drives acute inflammation and restricts expression durability independent of the DNA sequence. Recent studies have shown that incorporation of endogenous anti-inflammatory lipids, such as nitro-oleic acid, into DNA-loaded LNPs can suppress cGAS-STING signaling, enabling prolonged transgene expression exceeding 1 month while preventing severe inflammatory toxicity [220]. This anti-inflammatory co-loading strategy represents a broadly applicable platform for improving the safety and durability of LNP-based DNA therapeutics.

While DNA-based modalities offer the advantage of sustained expression and large cargo capacity, their reliance on nuclear delivery, limited tunability, and widespread CNS distribution presents significant challenges for translational purposes. These constraints have catalyzed increasing interest in RNA-based genome engineering approaches offering complementary advantages in safety, dosing, and flexibility to control protein and gene expression (Table 2). Together, DNA-based and RNA-based modalities illustrate the versatility of nucleic acid-driven interventions for the delivery of therapeutic proteins for CNS diseases. However, their clinical translation remains constrained, and innovation in LNP engineering will be critical to further enhance this as a gene therapy platform.

**Table 2:** Comparative design considerations for gene therapy modalities. This table summarizes key biological and delivery-related factors influencing the use of DNA, mRNA, regulatory RNAs, and CRISPR interventions relevant to central nervous system targeting

Design consideration	DNA	mRNA	Regulatory RNAs (ASO, siRNA, miRNA)	CRISPR
Expression duration	Sustained	Transient	Transient	Sustained or long term
Nuclear entry requirement	Required	Not required	Not required	Required
Redosing flexibility	Limited	High	High	Limited
Genomic alteration risk	None	None	None	Present
Safety profile	Moderate	High	High	Context dependent
Cargo capacity	High	Moderate	Low	Moderate to high

*ASO* antisense oligonucleotide, *CRISPR* clustered regularly interspaced short palindromic repeats, *miRNA* microRNA, *mRNA* messenger RNA, *siRNA* small interfering RNA

### Packaging DNA and RNA-Based Therapies in LNPs

Lipid nanoparticles have transformed the prospects of nucleic acid-based therapeutics, overcoming previous limitations associated with delivery and stability in cellular uptake, ushering in an age of novel vaccine therapeutics [217]. Despite these advances, delivery into the CNS remains a bottleneck as current clinical strategies rely on intrathecal and intracerebroventricular administration to bypass the BBB [239]. Thus, a growing interest in engineering BBB-penetrable LNPs capable of delivering diverse RNA and DNA modalities is essential to advance CNS disorder treatments. For example, researchers engineered an LNP with vascular cell adhesion molecule 1 antibodies, a protein expressed in BECs in response to neuroinflammation [240]. The vascular cell adhesion molecule 1-targeted LNP was delivered in an acute ischemic stroke mouse model and achieved a 100-fold increase in accumulation within cerebral blood vessels when compared with non-targeted LNPs. Moreover, when loaded with interleukin-10 mRNA, researchers observed 62% reduction in infarct size, highlighting the therapeutic potential of precision RNA delivery. Continued optimization of LNP composition and payload design will be critical to efficiently package and safely deliver a diverse repertoire of gene therapies across a wide range of CNS disorders.

## Future Perspectives

The future of LNP delivery to the brain is positioned to transform CNS therapeutics through advances in gene editing, epigenetic modulation, precision pharmacology, and personalized dosing strategies. The convergence of multiple technological advances suggests that there will be substantial progress in addressing current limitations and expanding therapeutic applications. Next-generation LNPs will likely enable safe, efficient, in vivo delivery of complex gene editors and epigenetic regulators to precisely target neuronal or glial populations. Advanced gene editing approaches such as prime editing, base editing, and epigenome editing offer unprecedented precision in correcting disease-causing mutations or modulating gene expression patterns. Lipid nanoparticle delivery of these molecular tools could enable treatment of monogenic neurological disorders and/or age-related neurodegenerative diseases.

Rational design increasingly employs artificial intelligence-driven lipid and ligand libraries to maximize BBB penetration and cell-type specificity while minimizing off-target effects and immune activation. Machine learning algorithms trained on expanding datasets of LNP performance should help enable the prediction of optimal formulations for specific therapeutic applications. While employing these unsupervised methods will surely accelerate the development of LNP formulations, an understanding of the biological problems remains important to guide design efforts. Evidence that it is possible to optimize against apparently contradictory objectives, such as minimizing peripheral tissue uptake while maximizing endothelial uptake, has been demonstrated in principle with the identification of detargeted LNP formulations [93]. Central nervous system targeting is doubly challenging as therapeutic delivery to endothelial cells is generally not the ultimate objective. To target parenchymal cells with gene therapy, an LNP must remain intact during transcytosis and subsequently fuse with neurons or glia. It can be imagined that multi-functional LNP formulations would be required to detarget peripheral tissues, enable transcytosis of intact LNPs across the BBB, and selectively mediate post-BBB uptake and payload release in neurons or glia.

Emerging data support direct CNS routes, such as intrathecal and intracerebroventricular administration, for broad and durable brain distribution of LNPs, offering promising avenues especially for rare genetic and pediatric disorders requiring single-dose treatments. For systemic administration, safety remains a critical priority. Ongoing refinements in lipid chemistry and surface engineering aim to reduce immunogenicity, complement activation, and hepatic toxicity, enabling the long-term administration necessary for neurodegenerative conditions. The development of biodegradable lipid components, improved stealth coatings, and cell-specific targeting will be essential for enabling repeat dosing regimens required for chronic neurological disorders.

Currently, LNP-mediated delivery systems have been clinically validated to deliver a variety of therapeutic modalities. Strategies targeting peripheral tissues following systemic delivery, such as the liver and lung, have shown great promise in clinical trials. Moreover, pre-clinical studies have demonstrated the utility of LNPs functionalized for BBB permeability. To date, however, clinical trials validating CNS-targeting technologies for LNP therapeutics are lacking. These collective advances suggest that effective treatments for currently intractable neurological conditions may become a reality within the next decade.
